# Supplementary material for: Adsorption and Desorption of Immune-Modulating Substances by Aluminium-Based Adjuvants: An Overlooked Feature of the Immune-Stimulating Mechanisms of Aluminium-Based Adjuvants
Source: Int J Mol Sci. 2024 Nov 19;25(22):12399. doi: 10.3390/ijms252212399 (PMC11594729; doi:10.3390/ijms252212399)
Supplement: Supplementary file 1 [file ijms-25-12399-s001.zip › Suplemt mat resubmit.pdf]

# Supplementary material

**Figure S1** Induction of MHC expression by IFN-γ desorbed from Alhydrogel®
Histograms of THP-1 cells showing the expression of MHC after; (A) Addition of 37.5 ng IFN-γ into the culture medium, giving the concentration of 8.6 ng IFN-γ/ml, (B) Culture in medium without any addition of IFN-γ, (C) Addition of 32 µg Alhydrogel® containing at most 11.7 ng adsorbed IFN-γ, (D) Addition of 200 µg Alhydrogel® containing no adsorbed IFN-γ
The cells were cultured over-night, day 1, removed from the TC inserts and stained with antibodies against MHC class I and class II. New TC insert with cells were added to the previously used medium. The cells were cultured over-night, day 2, removed from the TC inserts and stained with antibodies against MHC class I and class II. (1) Staining with an isotype control antibody, (2) Cells at day 1 stained with an APC labelled antibody against human MHC class I, (3) Cells at day 2 stained with an APC labelled antibody against human MHC class I, (4) Cells at day 1 stained with a FITC labelled antibody against human MHC class II, (5) Cells at day 2 stained with a FITC labelled antibody against human MHC class II.
Black line showing and representing the peak fluorescence channel from cells cultured after the addition of 37.5 ng IFN-γ to the culture medium.

**Table S1**
Location of controls and cytokines on the array membrane
The spot on the upper left has the coordinate A1 and the spot on the lower right has the coordinate E20

| Coordinate | Target/control | Coordinate | Target/control |
|---|---|---|---|
| A1, A2 | Reference Spots | C7, C8 | IL-4 |
| A3, A4 | CCL1/I-309 | C9, C10 | IL-5 |
| A5, A6 | CCL2/MCP-1 | C11, C12 | IL-6 |
| A7, A8 | MIP-1α/MIP-1β | C13, C14 | IL-8 |
| A9, A10 | CCL5/RANTES | C15, C16 | IL-10 |
| A11, A12 | CD40 Ligand | C17, C18 | IL-12 p70 |
| A13, A14 | Complement C5/C5a | D3, D4 | IL-13 |
| A15, A16 | CXCL1/GROα | D5, D6 | IL-16 |
| A17, A18 | CXCL10/IP-10 | D7, D8 | IL-17A |
| A19, A20 | Reference Spots | D9, D10 | IL-17E |
| B3, B4 | CXCL11/I-TAC | D11, D12 | IL-18/IL-1F4 |
| B5, B6 | CXCL12/SDF-1 | D13, D14 | IL-21 |
| B7, B8 | G-CSF | D15, D16 | IL-27 |
| B9, B10 | GM-CSF | D17, D18 | IL-32a |
| B11, B12 | ICAM-1/CD54 | E1, E2 | Reference Spots |
| B13, B14 | IFN-γ | E3, E4 | MIF |
| B15, B16 | IL-1α/IL-1F1 | E5, E6 | Serpin E1/PAI-1 |
| B17, B18 | IL-1β/IL-1F2 | E7, E8 | TNF-α |
| C3, C4 | IL-1ra/IL-1F3 | E9, E10 | TREM-1 |
| C5, C6 | IL-2 | E19, E20 | Negative Control |

**Table S2.** Culture of THP-1 cells in the presence of added IFN-γ and Alhydrogel® containing adsorbed γ-IFN.
THP-1 cells cultured in tissue culture (TC) inserts and stained with antibodies against human MHC class I (anti HLA-ABC) and against MHC class II (anti HLA-DR, DP, DQ) after co-culture with various concentrations of directly added IFN-γ or Alhydrogel® containing adsorbed γ-IFN. Concentrations of IFN-γ in the culture medium were determined by ELISA.
Expression of MHC presented as the ratio between mean fluorescence intensity (MFI) after staining with labelled antibody against MHC from cells cultured in the presence of IFN-γ or Alhydrogel® and the MFI obtained after staining THP-1 cells cultured in TC insert and medium (R10 control). The Table shows representative results from one of three independent experiments performed

| Sample | Day 0 | | Day 1 | | | Day 2 | | |
|---|---|---|---|---|---|---|---|---|
| | Added IFN-γ (ng) in a total volume of 4ml | Medium conc[a] (pg/ml) | Ratio MFI sample / MFI R10 | | Medium conc[b] (pg/ml) | Ratio MFI sample / MFI R10 | | Medium conc (pg/ml) |
| | | | MHC-I | MHC-II | | MHC-I | MHC-II | |
| Medium control, R10 | 0 | 0 | 1.0 | 1.0 | 0 | 1.0 | 1.0 | 0 |
| Free IFN-γ | 7.5 | 1700 | 1.3 | 18.7 | 335 | 1.8 | 12.7 | 237 |
| Free IFN-γ | 37.5 | 8600 | 1.4 | 27.8 | 1639 | 1.8 | 24.6 | 1894 |
| 200 µg Alhydrogel® | 0 | 0 | 1.0 | 1.0 | 0 | 1.0 | 1.0 | 0 |
| 6.7 µg Alhydrogel®*IFN-γ | 2.5[c] | 266 | 1.3 | 9.7 | 103 | 1.6 | 5.0 | 84 |
| 32 µg Alhydrogel®*IFN-γ | 11.7[c] | 576 | 1.4 | 21.3 | 542 | 1.9 | 18.5 | 620 |

[a] Concentration determined by ELISA before addition of TC insert and first portion of THP-1 and thus adding an additional 1 ml to the well.
[b] Concentration determined by ELISA before addition of a new TC insert and second portion of THP-1 and thus adding an additional 1 ml to the well.
[c] Calculated assuming 100% recovery of Alhydrogel® after loading with IFN-γ and washing
