# Supplementary figures and images for: Adsorption and Desorption of Immune-Modulating Substances by Aluminium-Based Adjuvants: An Overlooked Feature of the Immune-Stimulating Mechanisms of Aluminium-Based Adjuvants
Source: Int J Mol Sci. 2024 Nov 19;25(22):12399. doi: 10.3390/ijms252212399 (PMC11594729; doi:10.3390/ijms252212399)

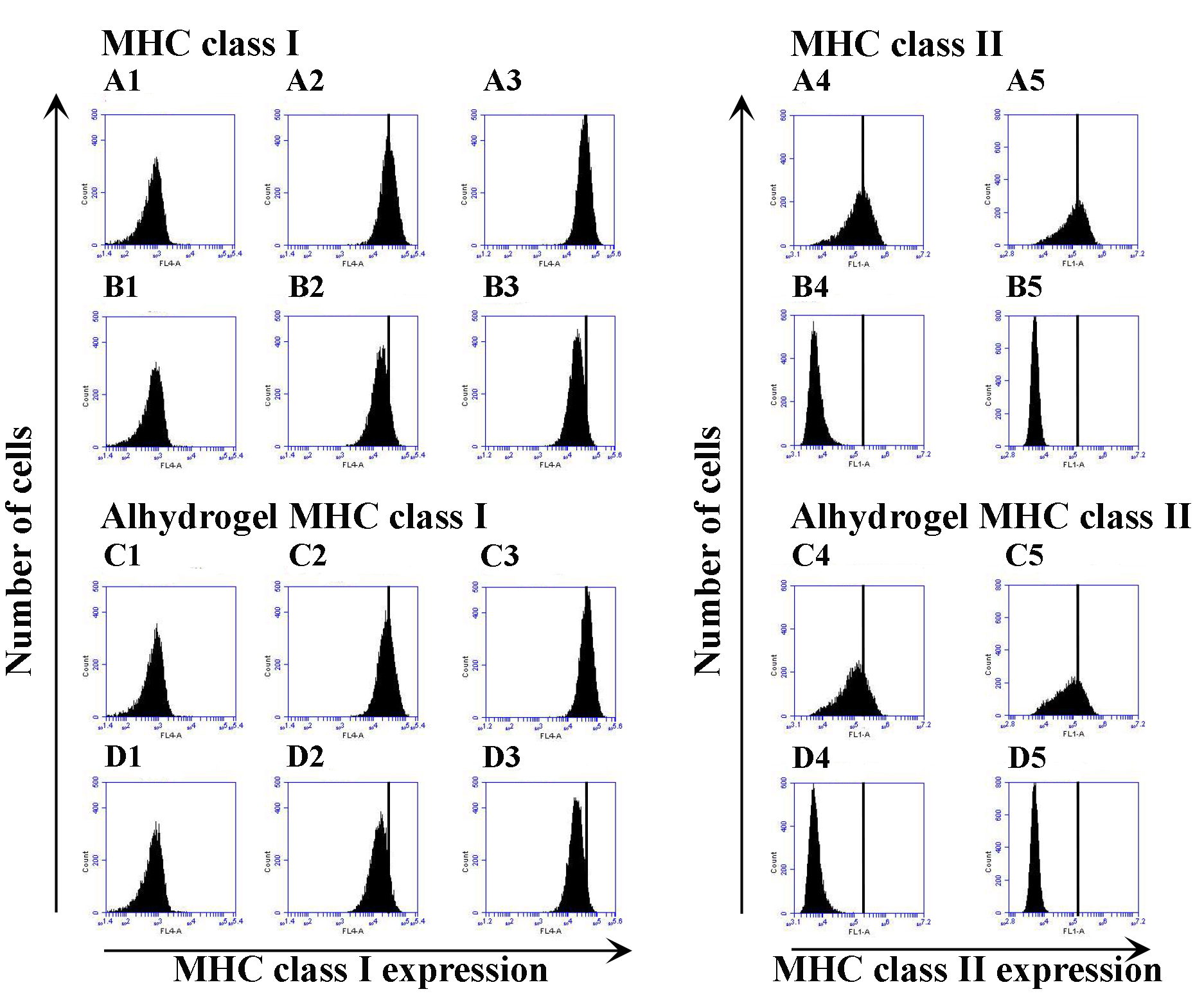

Supplement: Supplementary file 1 [file ijms-25-12399-s001.zip › Fig. S1. IJMS.jpg]
